# Heat transfer and pressure drop characteristic research of sine wavy flying-wing fins

**DOI:** 10.1038/s41598-023-42872-x

**Published:** 2023-09-20

**Authors:** Long Miao, Rui Wan, Hua-wei Wu, Zhen Liu, Shang-shun Wang

**Affiliations:** 1 School of Mechanical and Electrical Engineering, Xuzhou University of Technology, Jiangsu, 221000 China; 2 Hubei Key Laboratory of Power System Design and Test for Electrical Vehicle, Hubei University of Arts and Science, Xiangyang, 441053 China; 3 Shenzhen Shannon IoT Technologies LLC, Shenzhen, 518101 Guangdong China

**Keywords:** Mechanical engineering, Fluid dynamics

## Abstract

In recent years, heat transfer enhancement of heat exchange equipment has attracted more and more attention. In this paper, the heat transfer and pressure drop characteristics of sine wavy flying-wing fins are studied by numerical method. The objective is to improve the integrated heat transfer and pressure drop performance of sine wavy flying-wing fins. The degrees of freedom of fin sizes include fin pitch to fin height ratio *f*_p_/*f*_h_, fin height to fin wavelength ratio *f*_h_/*W*, fin amplitude to fin pitch ratio *2A*/*f*_p_ and fin inclined angle *α*. The results show that among the calculated 17 flying-wing fins, the optimal values of *f*_p_/*f*_h_, *f*_h_/*W*, *2A*/*f*_p_, and *α* are 0.5, 0.4, 1.9 and 70° respectively. The optimized SWFWF simulation model is established, and the average *JF* factor is 1.307, which is about 10.9% higher than that of Fin 05 (JF = 1.18). Multiple linear regression is used to obtain the correlations of flow and heat transfer characteristics of flying-wing fins. The average deviation of the correlations for *j* and *f* are 0.85% and 4.9% respectively. The correlations can be used for the design and optimization of sine wavy flying-wing fins.

## Introduction

In recent years, with the growth of industrial demand, heat transfer enhancement of heat exchange equipment has attracted more and more attention ^1,2^. Heat transfer equipment usually consists of tube and fin, and the main thermal resistance of heat exchanger is usually found in the air side, therefore, it is an effective way to improve the overall heat transfer performance of heat exchangers by increasing heat transfer area and convective heat transfer coefficient of the air side. Jia ^3^ proposes an aluminum flying-wing finned tube. The flying-wing fins (FWF) adopt an integral processing molding technology, which completely eliminates the contact thermal resistance between the fin and the tube. Experimental results show that the heat transfer of FWF is enhanced while the flow resistance of which is lower when compared with louvered fin-tube. However, when compared with plate fin-tube, the friction factor of FWF is about 50% higher. Therefore it is necessary to further study the FWF in order to improve thermal–hydraulic performance.

**Figure 1:**
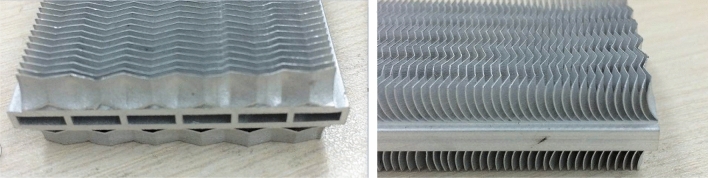
Heat transfer unit of FWF.

The corrugated fin is an effective way to enhance air-side heat transfer performance ^4,5^. Thus, the sine wavy-fins have been applied to FWF and the structural sizes of which is optimized in order to achieve better thermal–hydraulic performance. A part of the FWF is shown in Fig. 1.

At present, many research literatures on corrugated fins have been published. Several articles have analyzed in depth the heat transfer properties of corrugated fins of plate-fin heat exchangers, and proposed empirical correlations applicable to different ranges. Dong et al. ^6^ experimentally researched traditional corrugated finned flat tube radiators. Based on experimental data from 16 different sizes of fins, multiple regression analysis was conducted to obtain experimental correlations for heat transfer and pressure drop of corrugated fins. Naresh et al. ^7^ summarized the experimental data of corrugated fins and offset fins in a series of published literature, unified the fin size into 8 dimensionless quantities, and fitted the calculation correlation of the Colburn *j* factor and friction factor *f* for a wide range (120–10,000) of Reynolds numbers. The maximum deviation between the correlation calculation results and the experimental data of Kays and London ^8^ was within ± 5%. Siddhartha and Rath ^9,10^ studied the natural convection of wavy fins outside the horizontal tube, and analyzed the augmentation in momentum and heat transfer characteristics of wavy fins. The results showed that the heat transfer performance of corrugated fins is better than that of straight fins when the Ra number is high. Wan et al. ^11,12^ studied the thermal–hydraulic characteristics of wavy fins in plate-fin heat exchangers under negative gauge pressure environment through experiments and simulations, and proposed calculation correlations for the *j* factor and *f* factor. Miao et al. ^13,14^ conducted experimental and numerical simulation studies on FWF, compared and analyzed the heat transfer performance of FWF and traditional corrugated fins, and verified the superiority of FWF. Lin et al. ^15^ compared the performance of offset fins and flying-wing fins on the condenser of condensing dryers, and the results showed that FWF performed better than offset fins and the slit fins that came with dryers, and could improve the EU energy efficiency level of dryers from C to B.

Some researchers have improved the corrugated fins by adding vortex generators to enhance heat transfer. On the basis of the straight fin, Li et al. ^16^ added wavy ribs and carried out simulation studies. It was showed that Nu is increased by 40%, the inserted wavy rib can efficiently improve the heat transfer performance. Xue et al. ^17^ carried out simulation research on corrugated fins in plate-fin heat exchanger, and proposed three improved corrugated fins, perforated corrugated fins, staggered corrugated fins and discontinuous corrugated fins. The results showed that perforation on corrugated fins can enhance eddy current, promote fluid mixing, effectively improve heat transfer performance, and obtain a maximum performance evaluation criteria (PEC) of up to 1.24. Luo et al. ^18^ added a vortex generator to the corrugated fin, and the influence of the corrugated fin corrugation angle and the attack angle of the vortex generator were studied by numerical simulation, and the Nu, friction factor f and performance coefficient JF of different fins were compared and analyzed. The vortex generator increased the JF of the corrugated fins by up to 26.4%. Mohanta et al. ^19^ added slit fins to round tube corrugated fins to form hybrid slit wavy (HSW) fins, and simulated and analyzed the flow and heat transfer characteristics using commercial computational fluid dynamics software. Compared with the basic fins, the heat transfer of HSW fins is enhanced by 20–39%, the pressure drop is increased by 20–38%, and the area goodness factors of HSW fins are increased by 4%. Chimres et al. ^20^ studied the round tube corrugated fin heat exchanger of the air conditioning condenser by experimental and simulation means, and added rectangular winglets to the corrugated fins to obtain the optimal size of rectangular winglets. Compared with the original fin, factor JF of the wavy fin with winglets is 5.4 ~ 14.6% higher. Wu et al. ^21^ studied the perforated corrugated fins of heat pump air conditioning heat exchangers, simulated and analyzed the influence of opening on the heat transfer performance of the corrugated fins, obtained the optimal geometric size of the perforated fins, and experimentally verified the heat transfer enhancement performance of the perforated corrugated fins under frosting and non-frost conditions, which were improved by 4.1% and 8.9%, respectively.

The genetic algorithm optimization that has emerged in recent years has also been applied to the structural optimization of corrugated fins. Cui and Song ^22^ established a calculation model for corrugated fins based on the heat transfer and pressure drop correlations proposed by Qasem ^23^. Using genetic algorithm, the corrugated fin size optimization was carried out with minimum modified entropy generation number and maximum effectiveness *ε* as the objective functions. The results verified the effectiveness of modified entropy generation number as an objective function for the comprehensive performance optimization of plate-fin heat exchanger.

At present, the published research on corrugated fins is mainly aimed at conventional plate-fin heat exchangers, while FWF related research is not yet sufficient. In this paper, the sine wavy flying-wing fin (SWFWF) is studied, the influence of dimensionless fin size parameters on its heat transfer and pressure drop characteristics is analyzed, and the size parameters of FWF are optimized to obtain better thermal–hydraulic performance. Four dimensionless parameters are selected to represent the fin size, including fin pitch to fin height ratio *f*_p_/*f*_h_, fin height to fin wavelength ratio *f*_h_/*W*, fin amplitude to fin pitch ratio *2A*/*f*_p_ and fin inclined angle *α*. A total of 17 sets of fins with different sizes are designed, and the air-side thermal–hydraulic characteristics are simulated. Then, the influence of dimensionless numbers on the flow field and temperature field is analyzed. The empirical correlations for heat transfer and flow resistance are fitted by a total of 140 data points. Finally, the optimized SWFWF simulation model is established, and the superiority of its flow and heat transfer performance is verified by comparison with the original fin model. The empirical correlations established in this article can be used for the design and optimization of SWFWF corrupted fins The SWFWF performance optimization method adopted in the article is practical and feasible.

## Fin sizes

**Figure 2:**
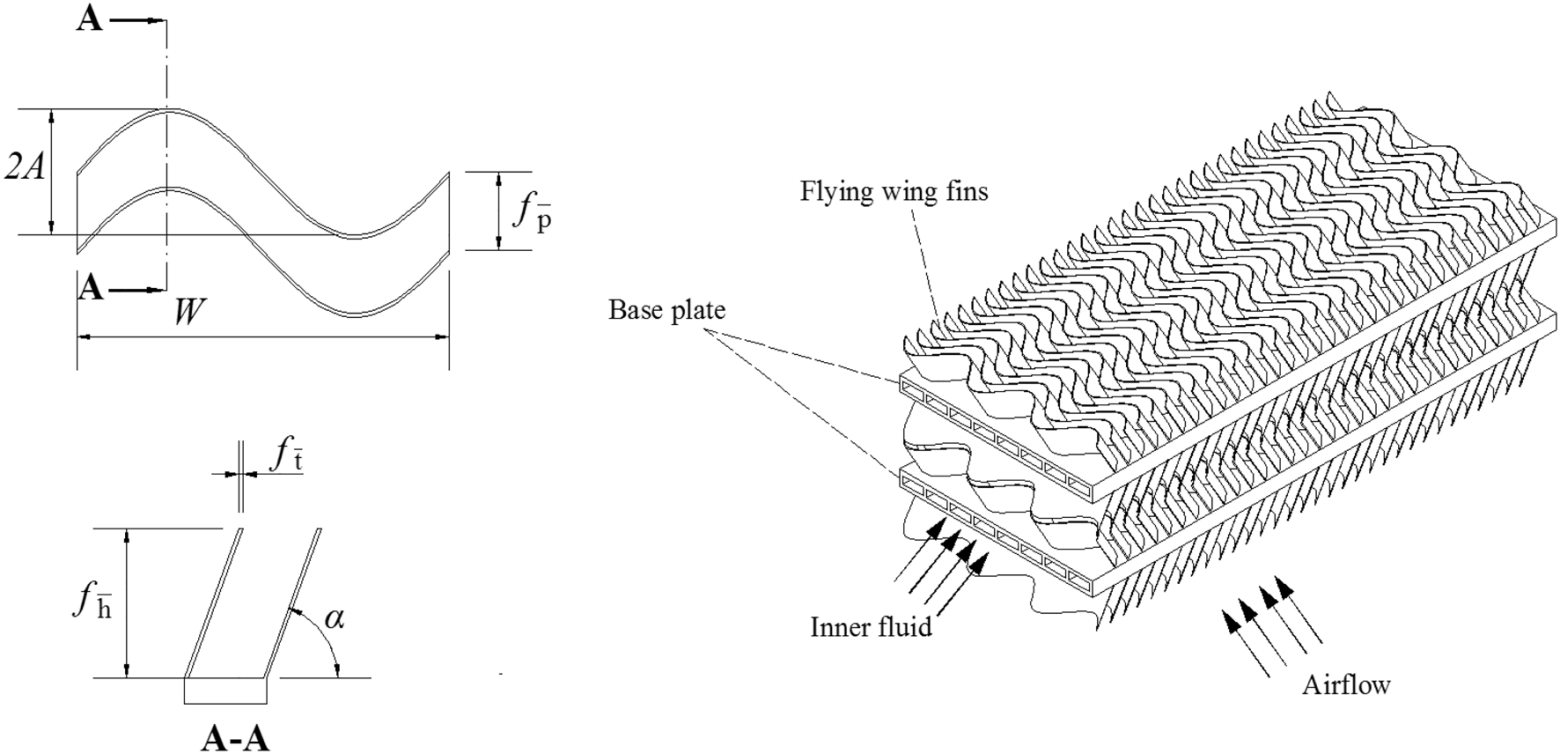
Basic unit of sine wavy flying-wing fin.

The object of this study is a basic unit of sine wavy flying-wing fin, as shown in Fig. 2. The main variables include fin pitch *f*_p_, fin height *f*_h_, fin wavelength *W*, fin amplitude *2A* and fin inclination angle *α*. In this study, fin thick *f*_*t*_ remains constant, 0.3 mm.

Four dimensionless parameters are selected to represent the fin size, including fin pitch to fin height ratio *f*_p_/*f*_h_, fin height to fin wavelength ratio *f*_h_/*W*, fin amplitude to fin pitch ratio *2A*/*f*_p_ and fin inclined angle *α*. The *f*_p_/*f*_h_ influences the hydraulic diameter of the flow channel, which in turn affects the Reynolds number at the same oncoming wind speed, and affects the flow field and temperature field between the fins. The *f*_h_/*W* affects the resistance of the fin flow channel. The *2A*/*f*_p_ affects the boundary layer in the flow channel. The *α* affects the heat transfer area and affects the heat transfer and flow resistance between the fins. The range of variation of each parameter is: 0.1 ≤ *f*_p_/*f*_h_ ≤ 0.5, 0.3 ≤ *f*_h_/*W* ≤ 0.5, 1.5 ≤ *2A*/*f*_p_ ≤ 1.9 and 50° ≤ *α* ≤ 80°. The detailed parameters of each fin are shown in Table 1. There are 17 groups of different fin sizes.

**Table 1:** Parameters of fins.

No	*f*_p_/*f*_h_	*f*_h_/*W*	*2A*/*f*_p_	*α* (°)	*f*_h_ (mm)	*f*_p_ (mm)	*2A* (mm)	*W* (mm)
1	0.3	0.4	1.7	70	5.6	1.68	2.856	14
2	0.1	0.4	1.7	70	5.6	0.56	0.952	14
3	0.2	0.4	1.7	70	5.6	1.12	1.904	14
4	0.4	0.4	1.7	70	5.6	2.24	3.808	14
5	0.5	0.4	1.7	70	5.6	2.8	4.76	14
6	0.3	0.3	1.7	70	4.2	1.26	2.142	14
7	0.3	0.35	1.7	70	4.9	1.47	2.499	14
8	0.3	0.45	1.7	70	6.3	1.89	3.213	14
9	0.3	0.5	1.7	70	7	2.1	3.57	14
10	0.3	0.4	1.5	70	5.6	1.68	2.52	14
11	0.3	0.4	1.6	70	5.6	1.68	2.688	14
12	0.3	0.4	1.8	70	5.6	1.68	3.024	14
13	0.3	0.4	1.9	70	5.6	1.68	3.192	14
14	0.3	0.4	1.7	80	5.6	1.68	2.856	14
15	0.3	0.4	1.7	75	5.6	1.68	2.856	14
16	0.3	0.4	1.7	60	5.6	1.68	2.856	14
17	0.3	0.4	1.7	50	5.6	1.68	2.856	14

## Modeling methodology

### Mathematical formula

The flow state of air between the fins can be expressed by the Reynolds number ^24^.

1 $${\text{Re}} = \frac{{ud_{h} }}{\nu }$$

In which *u* represents the velocity of air (m s^−1^), *d*_h_ represents the hydraulic diameter (m), and *ν* represents kinematic viscosity of air (m^2^ s^−1^).

The expression of hydraulic diameter *d*_h_ is:

2 $$d_{h} = \frac{{4A_{c} }}{{L_{c} }} = \frac{{2f_{p} f_{h} }}{{f_{p} + {{f_{h} } \mathord{\left/ {\vphantom {{f_{h} } {\sin \alpha }}} \right. \kern-0pt} {\sin \alpha }}}}$$

In which *A*_c_ is the cross sectional area of channel between the fins, *L*_c_ represents the flow wetting perimeter.

The thermal–hydraulic performance of flying-wing fin can be expressed by Colburn *j* factor and Fanning friction factor *f* respectively. The expressions for *j* factor and *f* factor are ^24^ :

3 $$j = \frac{Nu}{{\Pr^{{{1 \mathord{\left/ {\vphantom {1 3}} \right. \kern-0pt} 3}}} \cdot {\text{Re}} }}$$

4 $$f = \frac{{d_{h} }}{4L} \cdot \frac{\Delta P}{{\left( {{1 \mathord{\left/ {\vphantom {1 2}} \right. \kern-0pt} 2}} \right)\rho_{air} u^{2} }}$$

In which:

5 $${\text{Nu}} = \frac{{h_{air} d_{h} }}{{\lambda_{air} }}$$

6 $$\Pr = \frac{{\mu c_{p,air} }}{{\lambda_{air} }}$$

In Eq. (4), inlet and exit contraction and expansion loss are ignored. The air properties are the average value. The local convective heat transfer coefficient and local Nusselt number are calculated as follows:

7 $$h_{air,x} = \frac{{q_{x} }}{{A_{x} \left( {T_{w,x} - T_{air,x} } \right)}}$$

8 $$Nu_{x} = \frac{{h_{air,x} d_{h} }}{{\lambda_{air} }}$$

### Conservation equations

The main assumptions in this simulation calculation are as follows:

1. The air flow in flying-wing fins is steady state and incompressible laminar flow;
2. Ignore thermal radiation and natural convection;
3. Throughout the simulation, the temperature at the bottom of the fin area, i.e. inside the tube, remains constant;

The conservation equations are as follows:

Continuity equation:

9 $$\frac{\partial }{{\partial x_{i} }}\left( {\rho_{m} u_{i} } \right) = 0$$

Momentum conservation equation:

10 $$\frac{\partial }{{\partial x_{i} }}\left( {\rho_{m} u_{i} u_{k} } \right) = \frac{\partial }{{\partial x_{i} }}\left( {\mu \frac{{\partial u_{k} }}{{\partial x_{i} }}} \right) - \frac{\partial p}{{\partial x_{k} }}$$

Energy conservation equation:

11 $$\frac{\partial }{{\partial x_{i} }}\left( {\rho_{m} u_{i} T} \right) = \frac{\partial }{{\partial x_{i} }}\left( {\frac{{\lambda_{air} }}{{c_{p} }}\frac{\partial T}{{\partial x_{k} }}} \right)$$

### Meshing and numerical methods

**Figure 3:**
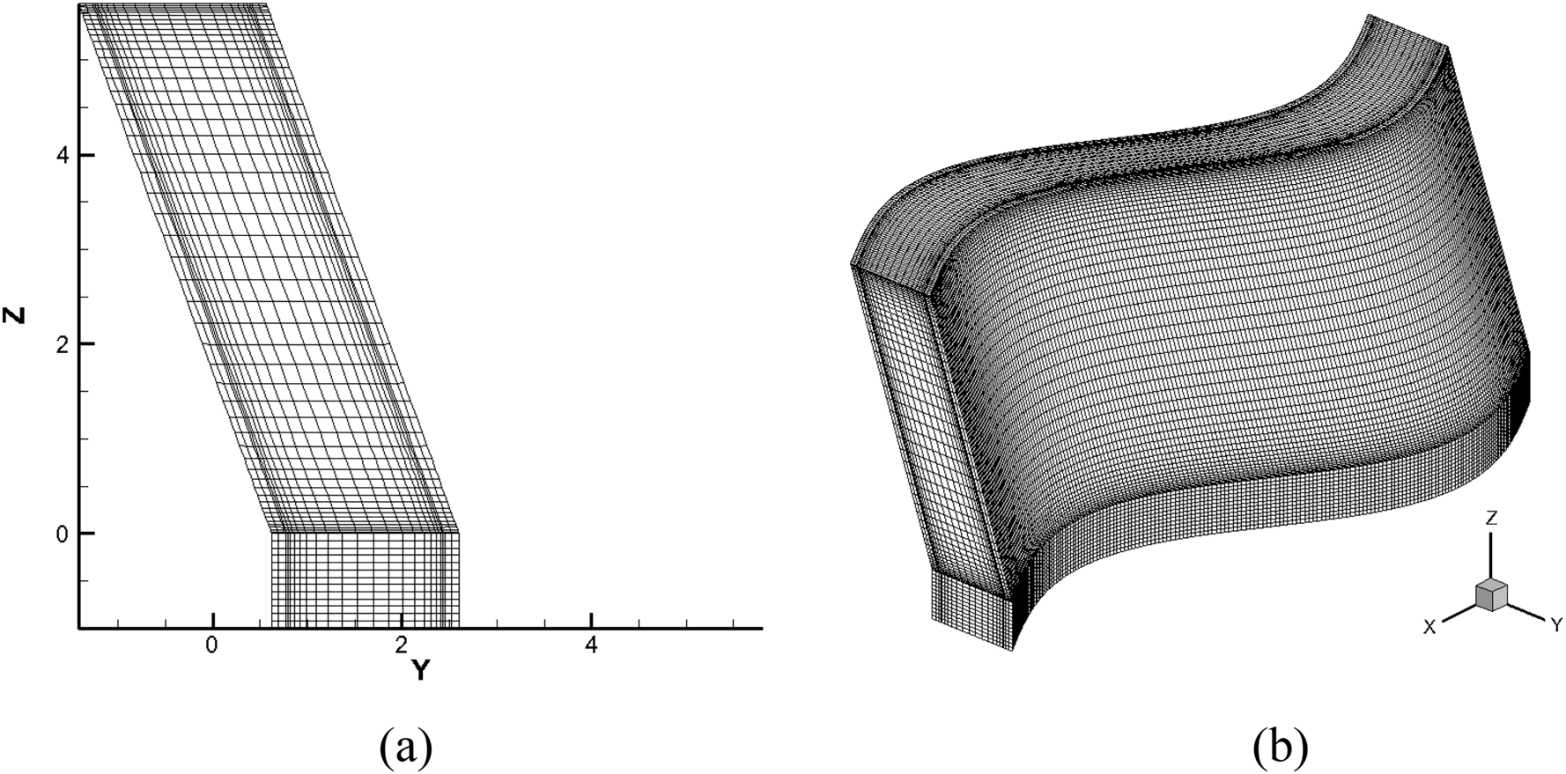
The mesh division. (**a**) local grid; (**b**) overall mesh.

The mesh division adopts hexahedral structured grids, which can improve the quality of the grid and reduce computational time. The mesh near the surface area of the fin is refined. The overall mesh and mesh near the wall are shown in Fig. 3.

**Figure 4:**
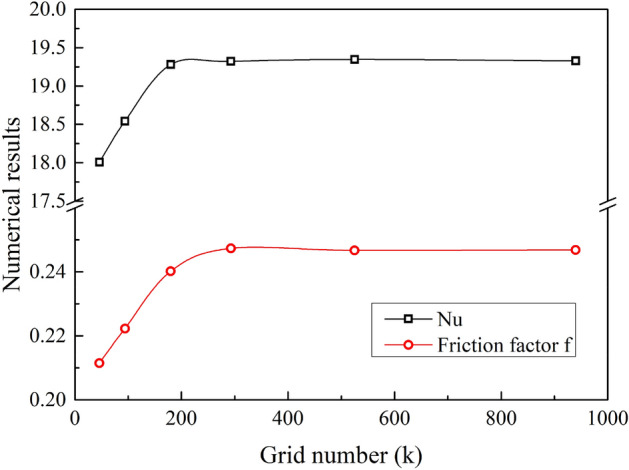
Mesh independence.

The calculation area is divided into different numbers of grids, and the number of grids is 46,000 ~ 940,000. As shown in Fig. 4, when the number of grids is higher than 293,000, the calculation results change less than 0.5%, so after the number of grids is higher than 293,000, the calculation result is independent of the grid number. The hexahedral mesh size is 0.08mm. The minimum orthogonal quality of the mesh is 0.803, and the maximum aspect ratio is 3.181.

According to actual condition, the maximum Reynolds number in the channel of the fins is 2000, which can be considered as laminar flow. The SIMPLE algorithm is implemented for coupling pressure and velocity. The second order upwind discretization scheme was applied to discretization of convective term. The convergence criterion for continuity is 10^–3^, the velocity term is 10^–6^, and the energy equation is 10^–8^.

### Computational domain and boundary conditions

**Figure 5:**
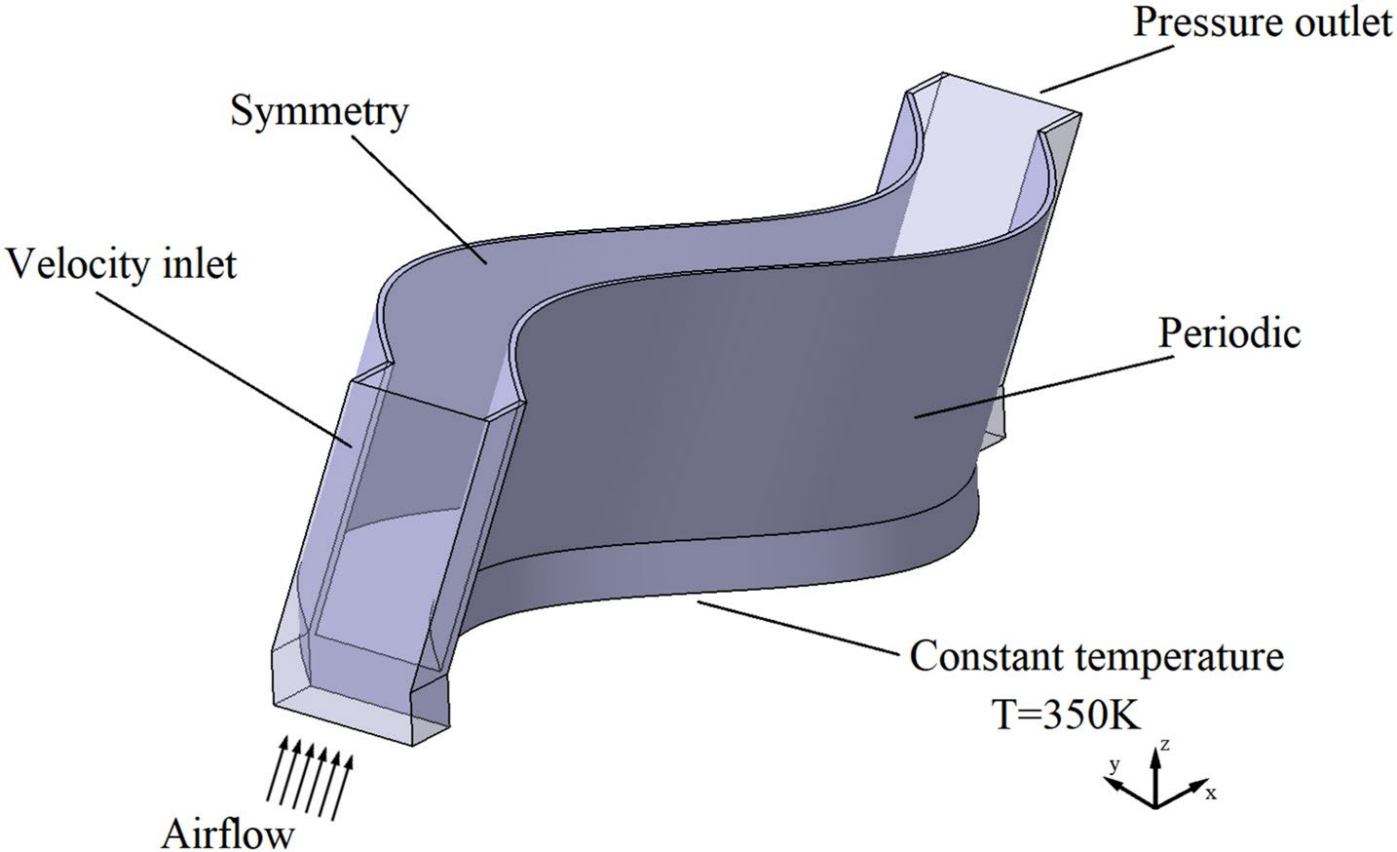
Computational domain and boundary conditions.

Figure 5 shows the simulation calculation area. The cold air flows forward along the X axis, is heated by the high-temperature fins on both sides, and then flows out of the flow channel between the fins. In order to ensure the accuracy of calculation, the computational region is extended. The regions upstream and downstream of the fin are lengthened by 1.5 and 5 times the fin wavelength, respectively, to obtain a more uniform flow rate distribution ^25^.

The main boundary conditions are shown in Fig. 5, and the specific parameter settings are shown in Table 2.The air flows into the fin area, and the bottom of the fin is a constant temperature surface. The inlet velocity is calculated from the Reynolds number and fin size. No-slip shear conditions are considered on all the solid walls. The bottom surface of the fin is primary heat transfer surface, so the wall temperature is considered as a constant. The fin surfaces are secondary heat transfer surfaces, and are considered as thermal coupling boundaries. Gravity is not considered in the simulation ^26,27^. The fin material is aluminum, the fluid is air, and the physical properties are shown in Table 3.

**Table 2:** Boundary conditions.

Boundary	Boundary conditions	Specific settings
Inlet	Velocity inlet	303.15 K, Re = 500 ~ 2000
Outlet	Pressure outlet	Gauge pressure 0Pa
Bottom surface of the fin	Wall	350K, constant temperature
Outer side of the fin	Periodic	Translational Periodic
Top surface of the fin	Symmetry

**Table 3:** Physical properties.

Material	Air	Aluminum
Density (kg m^−3^)	1.225	2,719
Specific heat (J kg^−1^ K^−1^)	1,006.43	871
Thermal conductivity (W m^−1^ K^−1^)	0.0242	202.4
Kinematic viscosity (Pa s)	1.46e^−5^	

### Computational model verification

**Figure 6:**
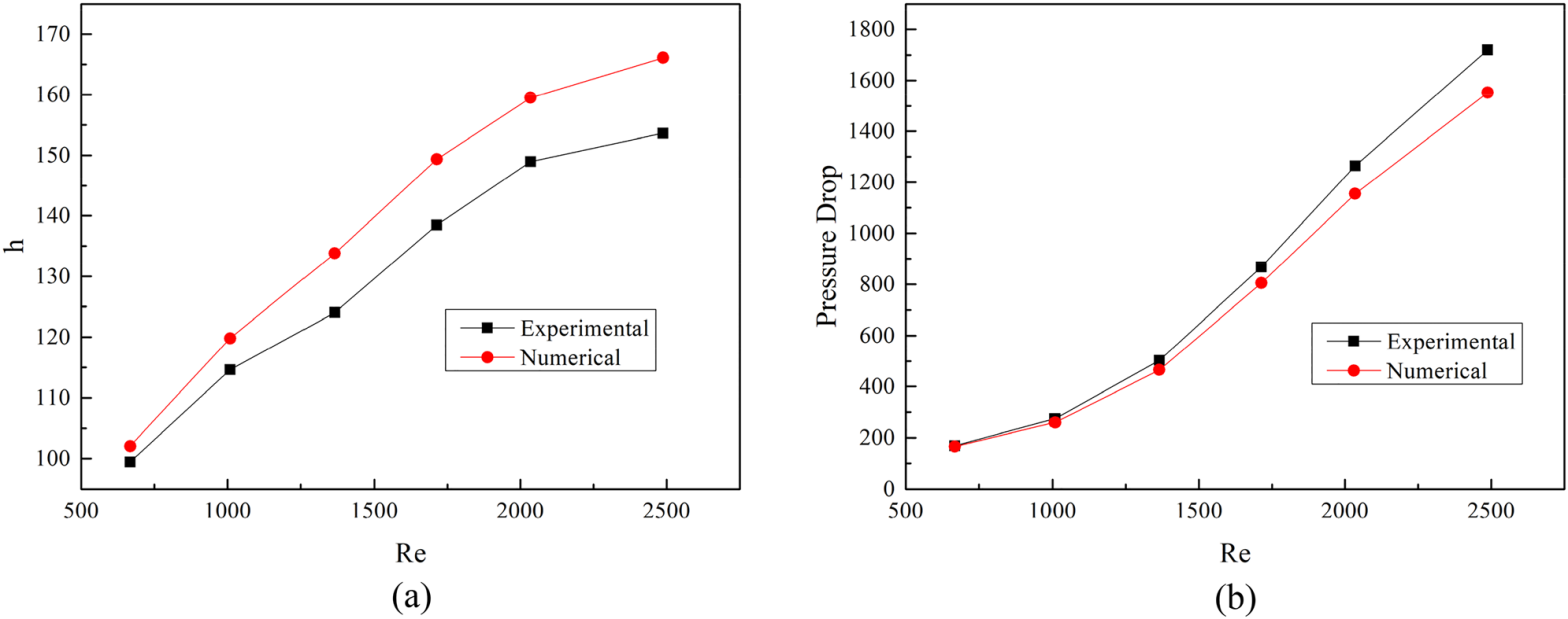
Computational model verification. (**a**) heat transfer coefficient *h*; (**b**) pressure drop Δ*P.*

Zhang et al. ^28^ conducted an experimental study of sharp corner wavy flying wing fins. This paper uses Zhang's experimental data to verify the simulation model. A complete fin model identical to the test piece is established, and the comparison of simulated results and experimental data is showed in Fig. 6.

The calculated results are in agreement with the experimental data. The maximum deviation and average deviation of *h* is 8.1% and 6.3%, respectively. The maximum deviation and average deviation of Δ*P* is 9.7% and 6.8%, respectively. Therefore, the numerical model and the calculation method established in this study are practical. Compared with experimental data, the simulation results of *h* are higher while the simulation results of ΔP are lower. The deviation between the numerical simulation and the experimental data increases with the increase of Re. The main reasons for the deviation are:

1. The uncertainty of experimental results. The relative deviation of convective heat transfer coefficient in the experiment is 4.89–5.5%, and that of friction coefficient is 1.39–8.8%.
2. Unevenness of the surfaces. The protuberance and burr in fin machining can increase forming resistance and cause overall friction. Those factors are difficult to consider in three-dimensional simulations.
3. Shape uncertainty. During the manufacturing process, the fins will inevitably undergo plastic deformation. However, in the simulation calculation, the ideal shape of the fins is considered.

In spite of the deviations between numerical simulation and experimental data, this degree of deviation is acceptable in engineering applications. Therefore, the heat transfer and pressure drop characteristics of SWFWF can be studied by the numerical model in this study.

## Results and discussion

Numerical simulations of 17 different sizes of SWFWF shown in Table 1 were conducted. The air in computational domain is in a laminar flow state, so each fin was calculated in Reynolds number range from 500 to 2000.

### Heat transfer

**Figure 7:**
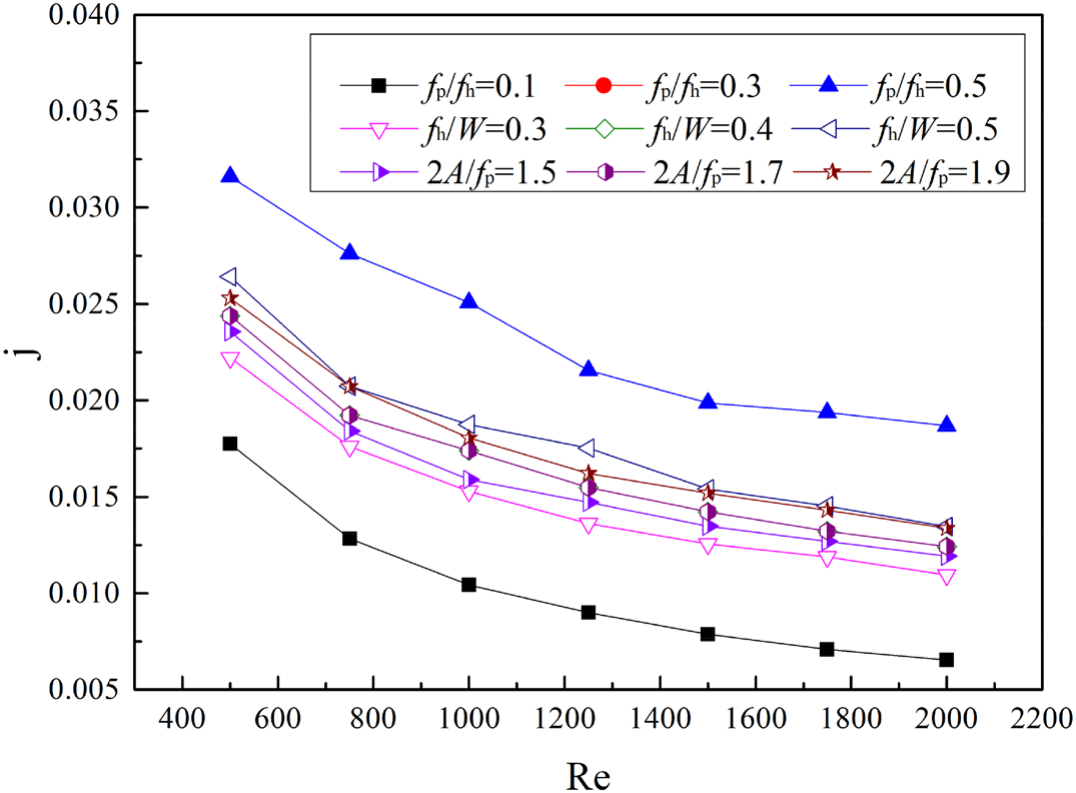
The j factor with different structural parameters.

Figure 7 shows the Colburn j factor of the fins with different structural parameters. The three curves in the figure, *f*_p_/*f*_h_ = 0.3, *f*_h_/*W* = 0.4, and *2A*/*f*_p_ = 1.7, coincide because they all correspond to the No.1 fin in Table 1 (The 3 dimensionless parameters, *f*_p_/*f*_h_, *f*_h_/*W*, and *2A*/*f*_p_ of No.1 fin are 0.3, 0.4, and 1.7, respectively). The j factor of all curves in the graph shows a decreasing trend as Re increases. When Re remains constant, an increase in *f*_p_/*f*_h_, *f*_h_/*W*, and *2A*/*f*_p_ will all lead to an increase in the j factor. The value of the j factor is smallest when *f*_p_/*f*_h_ = 0.1, which varies from 0.0178 to 0.0065. The j factor is largest when *f*_p_/*f*_h_ = 0.5, with an average of about 1.4 times that of *f*_p_/*f*_h_ = 0.1. When *f*_h_/*W* = 0.5, the j factor is about 1.22 times that of *f*_h_/*W* = 0.3 on average. When *2A*/*f*_p_ = 1.9, the j factor is about 1.12 times that of *2A*/*f*_p_ is 1.5.

**Figure 8:**
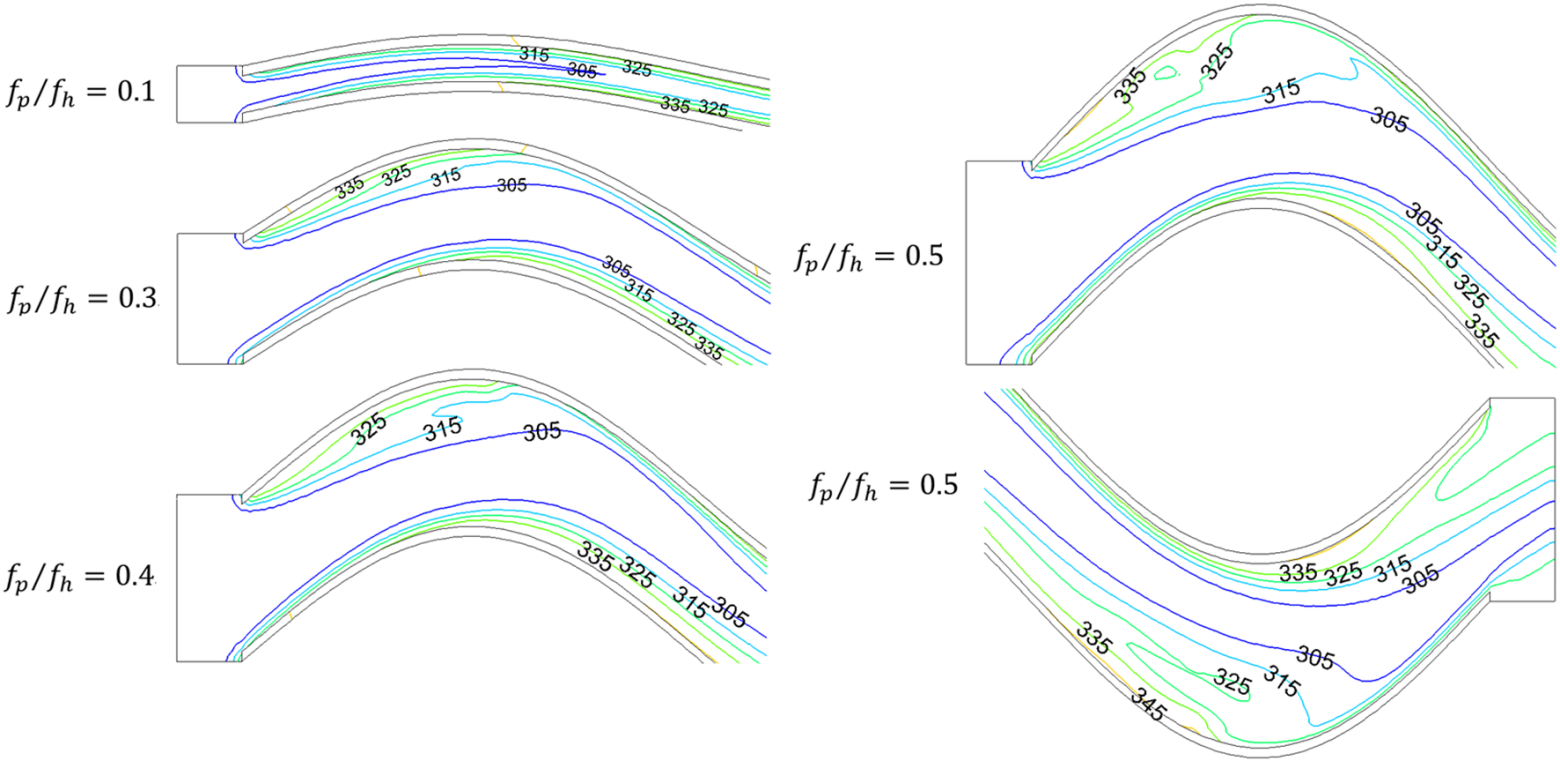
Temperature (K) distribution of different *f*_p_/*f*_h_.

The air-side temperature field with different *f*_p_/*f*_h_ is shown in Fig. 8. The air in the channel is gradually heated along the direction of flow. The air temperature gradient near the wall is much larger than that of the main stream, so the near wall region has important influence on the convective heat transfer. At the same time, it can be seen that the isotherms in the channel are almost parallel to the fin surface when the values of *f*_p_/*f*_h_ are 0.1 and 0.2. With the increase of *f*_p_/*f*_h_, the boundary layer is separated at the crest of the fin, where the isotherms change irregularly. When the value of *f*_p_/*f*_h_ is greater than 0.4, the irregularities of the isotherms are also observed at the trough of the fin. Therefore, the increase of *f*_p_/*f*_h_ results in the air boundary layer separation near the fins, which enhances the thermal convection in the channel.

### Pressure drop

**Figure 9:**
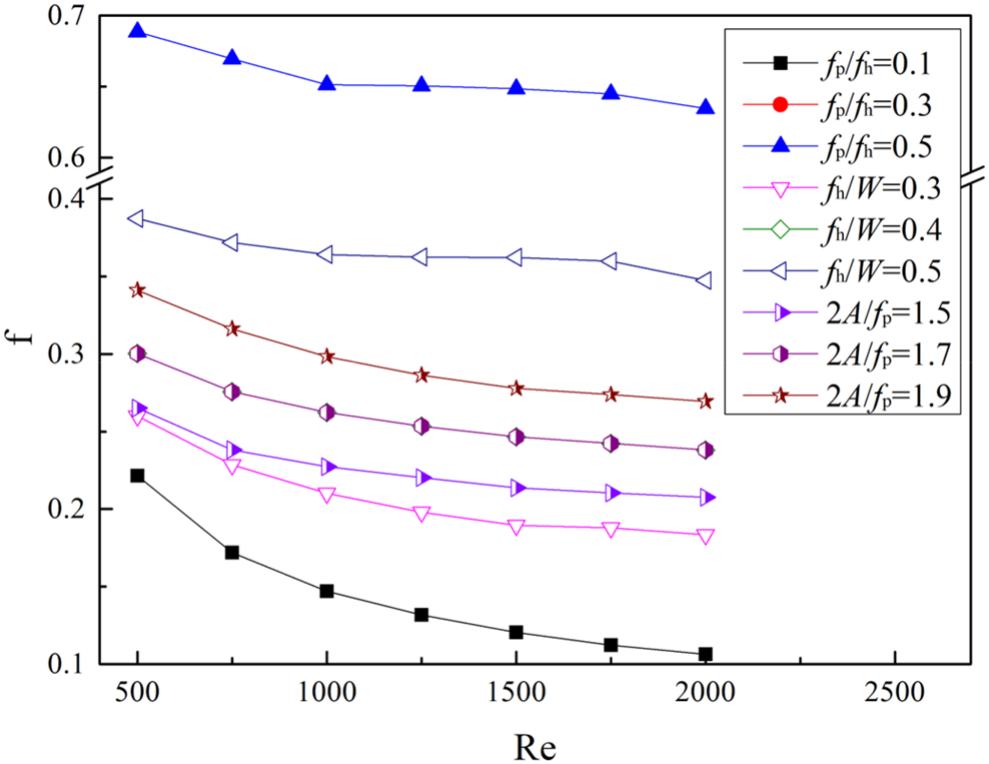
The f factor with different structural parameters.

The friction factor f of the fins is shown in Fig. 9. The three curves in the figure, *f*_p_/*f*_h_ = 0.3, *f*_h_/*W* = 0.4, and *2A*/*f*_p_ = 1.7, coincide because they all correspond to the No.1 fin in Table 1.The friction factor f decreases with the increase of Re. When *f*_p_/*f*_h_ = 0.1, the *f* factor is the lowest, which varies from 0.221 to 0.106 as Re increases from 500 to 2000. When other parameters are the same, the trend of *f* factor with the 3 dimensionless parameters is the same, all increasing with the increase of dimensionless parameters. The f factor is largest when *f*_p_/*f*_h_ = 0.5, with an average of about 3.7 times that of *f*_p_/*f*_h_ = 0.1. When *f*_h_/*W* = 0.5, the f factor is about 1.77 times that of *f*_h_/*W* = 0.3 on average. When *2A*/*f*_p_ = 1.9, the f factor is about 1.3 times that of *2A*/*f*_p_ is 1.5.

### JF factor

From the foregoing, it can be seen that the integrated heat transfer and pressure drop characteristics of SWFWF cannot be judged by the Colburn j factor or the friction factor *f* alone, so *JF* factor is introduced. The expression for the *JF* factor is as follows ^29^:

12 $$JF = \frac{{{{j_{i} } \mathord{\left/ {\vphantom {{j_{i} } {j_{0} }}} \right. \kern-0pt} {j_{0} }}}}{{\left( {{{f_{i} } \mathord{\left/ {\vphantom {{f_{i} } {f_{0} }}} \right. \kern-0pt} {f_{0} }}} \right)^{{{1 \mathord{\left/ {\vphantom {1 3}} \right. \kern-0pt} 3}}} }}$$

where the subscript *i* represents the fin parameter variable, which means *f*_p_/*f*_h_ here (*f*_p_/*f*_h_ = 0.1, 0.2, 0.3, 0.4, 0.5). Subscript 0 represents the fin in reference^25^ .

**Figure 10:**
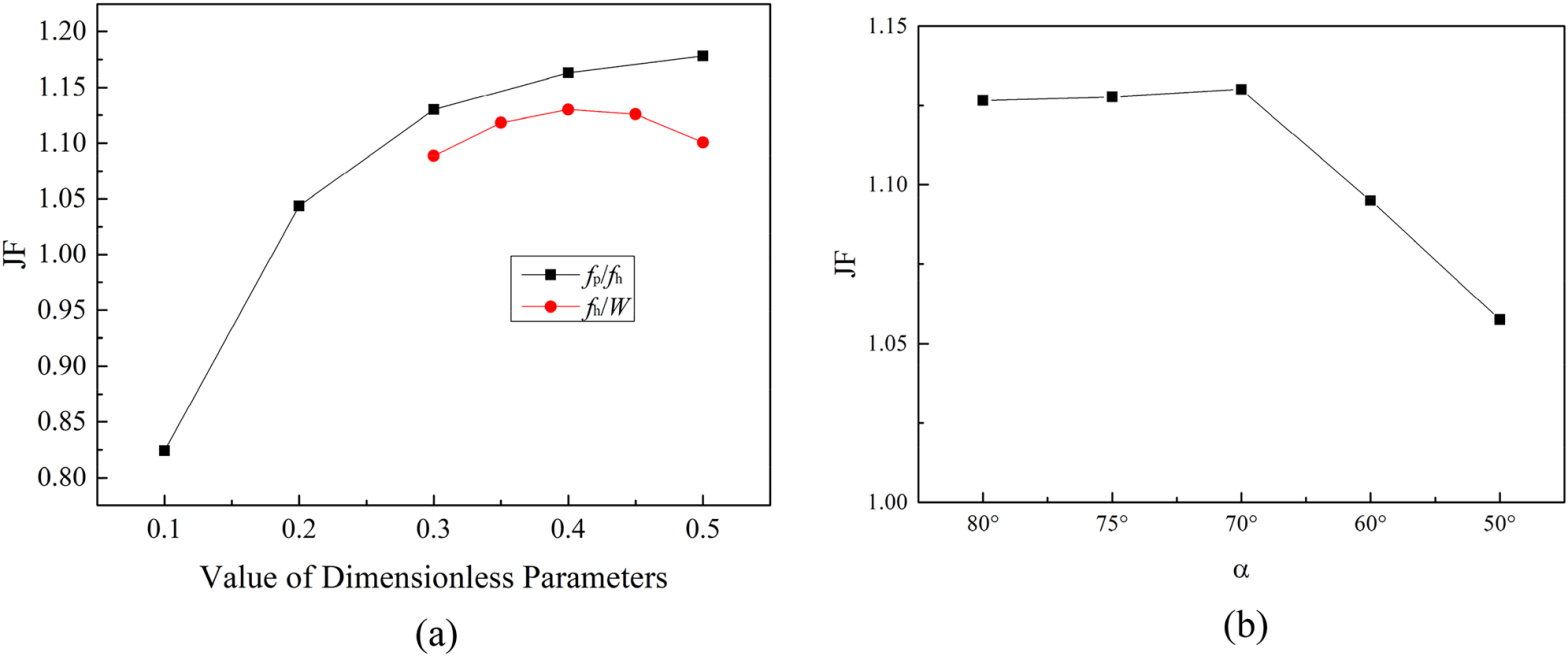
The *JF* factor under different parameters. (**a**) *f*_p_/*f*_h_, *f*_h_/*W*; (**b**) *α*.

The *JF* factor under different parameters is shown in Fig. 10. *JF* factor increases along with the increase of *f*_p_/*f*_h_, moreover, the gradient of JF decreases gradually. The value of *JF* factor is 0.82 when *f*_p_/*f*_h_ = 0.1, and *JF* reaches 1.18 when *f*_p_/*f*_h_ = 0.5. With the increase of *f*_h_/*W*, the *JF* factor increases first and then decreases. The *JF* factor reaches a maximum value of 1.13 when the value of *f*_h_/*W* is 0.4. As α increases, *JF* first increases and then decreases. The *JF* reaches a maximum value of 1.13 when *α* = 70°.

### Field synergy analysis

Field synergy theory states that the heat transfer intensity of convection is not only affected by fluid flow velocity, physical properties and fluid–solid temperature difference, but also depends on the synergistic effect between the fluid velocity field and the temperature field. When other conditions are consistent, the higher the degree of synergy between the temperature field and the velocity field, the higher the convective heat transfer intensity. The degree of coordination between the temperature field and the velocity field can be described by the field synergy angle ^30^.

The expression of the inner product of velocity and temperature gradient is:

13 $$\overrightarrow {U} \cdot grad\overrightarrow {T} = \left| {\overrightarrow {U} } \right| \cdot grad\left| {\overrightarrow {T} } \right| \cdot \cos \theta$$

**Figure 11:**
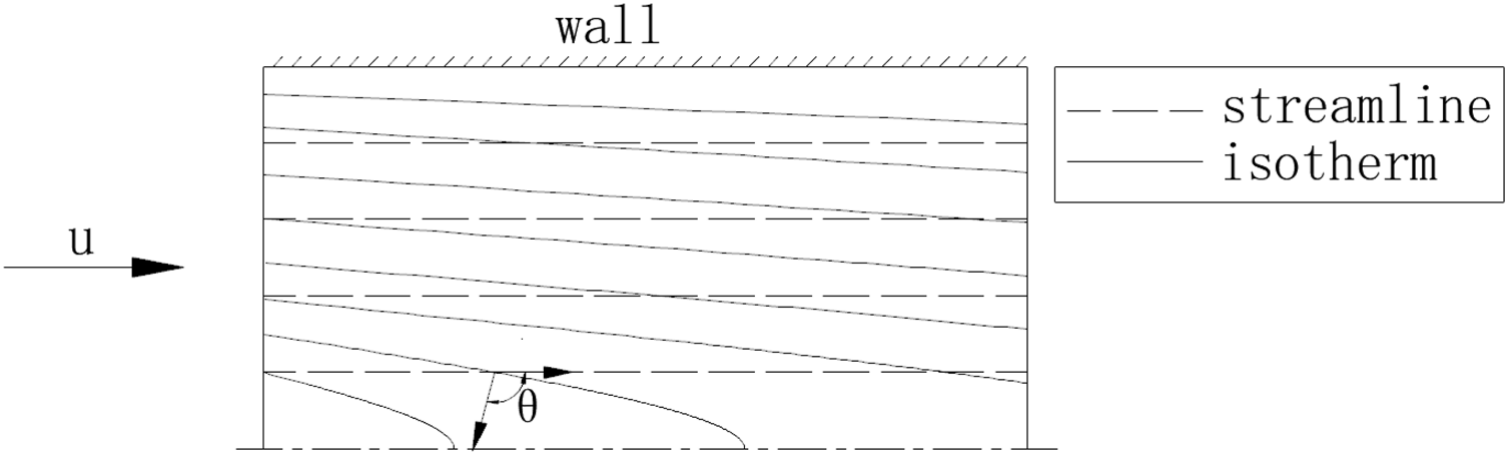
Schematic of the field synergy angle (FSA).

In the above equation, *θ* represents the angle between the velocity vector and the temperature gradient vector. This angle is also known as the Field Synergy Angle (FSA). Figure 11 shows the schematic of the field synergy angle.

The formulas below are local FSA and average FSA, respectively ^31,32^:

14 $$\theta_{i} = \cos^{ - 1} \frac{{\left| {u\frac{\partial T}{{\partial x}} + v\frac{\partial T}{{\partial y}} + w\frac{\partial T}{{\partial z}}} \right|}}{{\left| {\overrightarrow {U} } \right| \cdot \left| {grad\overrightarrow {T} } \right|}}$$

15 $$\theta_{m} = \frac{{\sum {\Delta C_{i} \theta }_{i} }}{{\sum {\Delta C}_{i} }}$$

In the above formula, *ΔC*_i_ represents the cell volume of each control volume, *θ*_i_ represents the FSA in a node, and *θ*_m_ represents the average FSA in the simulation calculation area.

**Figure 12:**
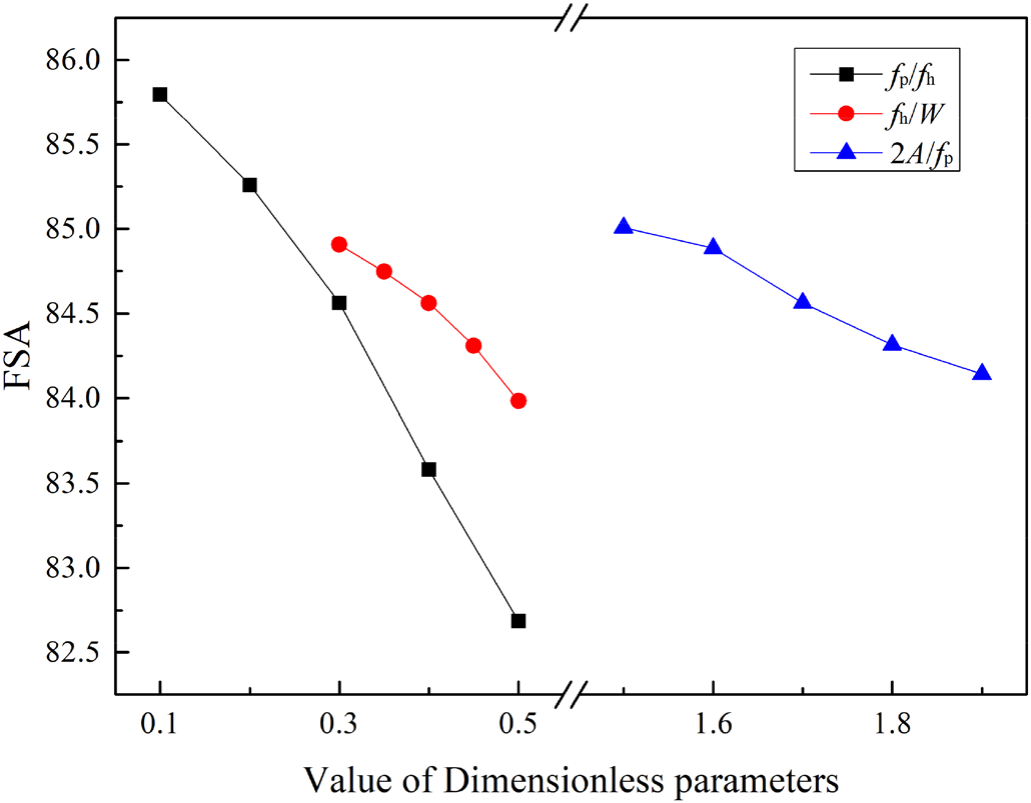
Average field synergy angle (FSA).

Figure 12 shows the average FSA under different structural parameters.

In Fig. 12, it is obvious that the FSA is on a downward trend with the increase of *f*_p_/*f*_h_, *f*_h_/*W* and *2A*/*f*_p_. Among the calculated 17 different sizes of SWFWF, the maximum value of the average FSA is 85.8 degrees, when *f*_p_/*f*_h_ = 0.1; the minimum value of average FSA is 82.7 degrees, when *f*_p_/*f*_h_ = 0.5. The variation range of average FSA is small, but it has obvious influence on comprehensive heat transfer and pressure drop performance. By changing the values of dimensionless parameters selected in this study, the angle between the temperature gradient and the velocity gradient can be changed, and then the comprehensive heat transfer and pressure drop performance of SWFWF can be affected.

### Empirical correlations

It can be seen from the above that each dimension parameter has different influence on the thermal–hydraulic performance of SWFWF. In order to comprehensively consider the influence of the above dimensionless parameters on heat transfer and flow resistance, it is necessary to propose an empirical correlation of the Colburn *j* factor and the friction factor *f*. Based on the numerical simulation results, the empirical correlation formula obtained by multiple linear regression method is as follows, including a total of 140 data points ^33^.

16 $$j = 1.17{\text{Re}}^{ - 0.493} \left( {\frac{{f_{p} }}{{f_{h} }}} \right)^{0.535} \left( {\frac{{f_{h} }}{W}} \right)^{0.399} \left( {\frac{2A}{{f_{p} }}} \right)^{0.452} \left( {\frac{\alpha }{90^\circ }} \right)^{0.132}$$

17 $$f = 4.59{\text{Re}}^{ - 0.186} \left( {\frac{{f_{p} }}{{f_{h} }}} \right)^{0.915} \left( {\frac{{f_{h} }}{W}} \right)^{1.11} \left( {\frac{2A}{{f_{p} }}} \right)^{1.16} \left( {\frac{\alpha }{90^\circ }} \right)^{ - 0.0248}$$

**Figure 13:**
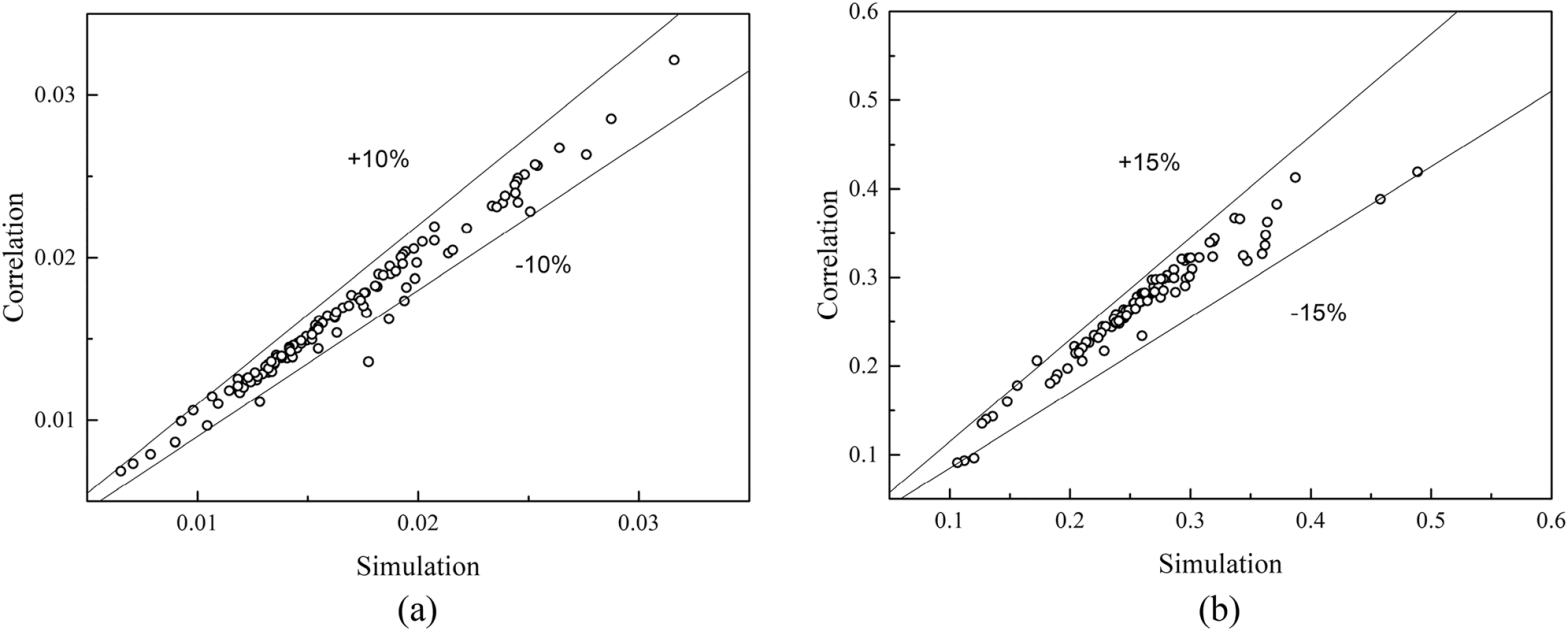
Comparison between simulation and correlations. (**a**) j factor; (**b**) f factor.

Figure 13 shows the calculation deviation of the empirical correlations. Equations (16) and (17) can predict 95% of the simulation results of *j* and *f*. The maximum deviation of *j* is less than 10%, and the maximum deviation of *f* is less than 15%. The equation for mean deviation and average deviation are Eq. (18) and Eq. (19) ^34^. The mean deviation of Eq. (16) and Eq. (17) are 2.5% and 7.2%, and the average deviations are 0.85% and 4.9%, respectively.

18 $$Average \, deviation = \frac{1}{N}\left( {\sum {\frac{\phi cor - \phi sim}{{\phi sim}}} } \right) \times 100\%$$

19 $$Mean \, deviation = \frac{1}{N}\left( {\sum {\frac{{\left| {\phi cor - \phi sim} \right|}}{\phi sim}} } \right) \times 100\%$$

### The optimized fins

Within the range of values in this article, the optimal values of *f*_p_/*f*_h_, *f*_h_/*W*, and *α* are 0.5, 0.4, and 70°, respectively. Therefore, the optimal values are used to establish the optimized fin, and simulation analysis is carried out. The optimal fin sizes established are shown in Table 4. No. 18 fin has one wavelength and No. 19 fin has three wavelengths.

**Table 4:** Parameters of the optimized fins.

No.	18	19
*f*_h_ (mm)	5.6	5.6
*f*_p_ (mm)	2.8	2.8
*2A* (mm)	5.32	5.32
*W* (mm)	14	14
*α* (°)	70	70
Number of waves	1	3

**Figure 14:**
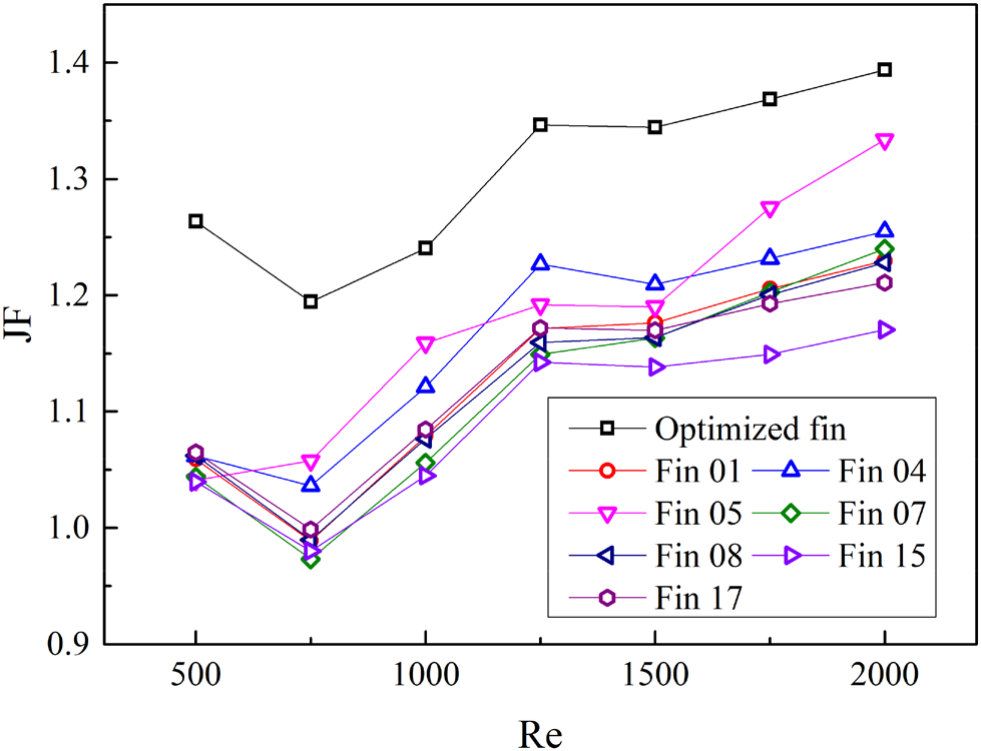
Change in JF factor of the optimized fin.

The variation of *JF* factor of No.18 fin with Re is shown in Fig. 14, and the simulation results of some other fins are also shown. The variation range of *JF* factor of the optimized fin is 1.19 ~ 1.39. The average *JF* factor is improved by approximately 10.9% compared to Fin 05. The optimized SWFWF's thermal–hydraulic performance has been improved.

**Figure 15:**
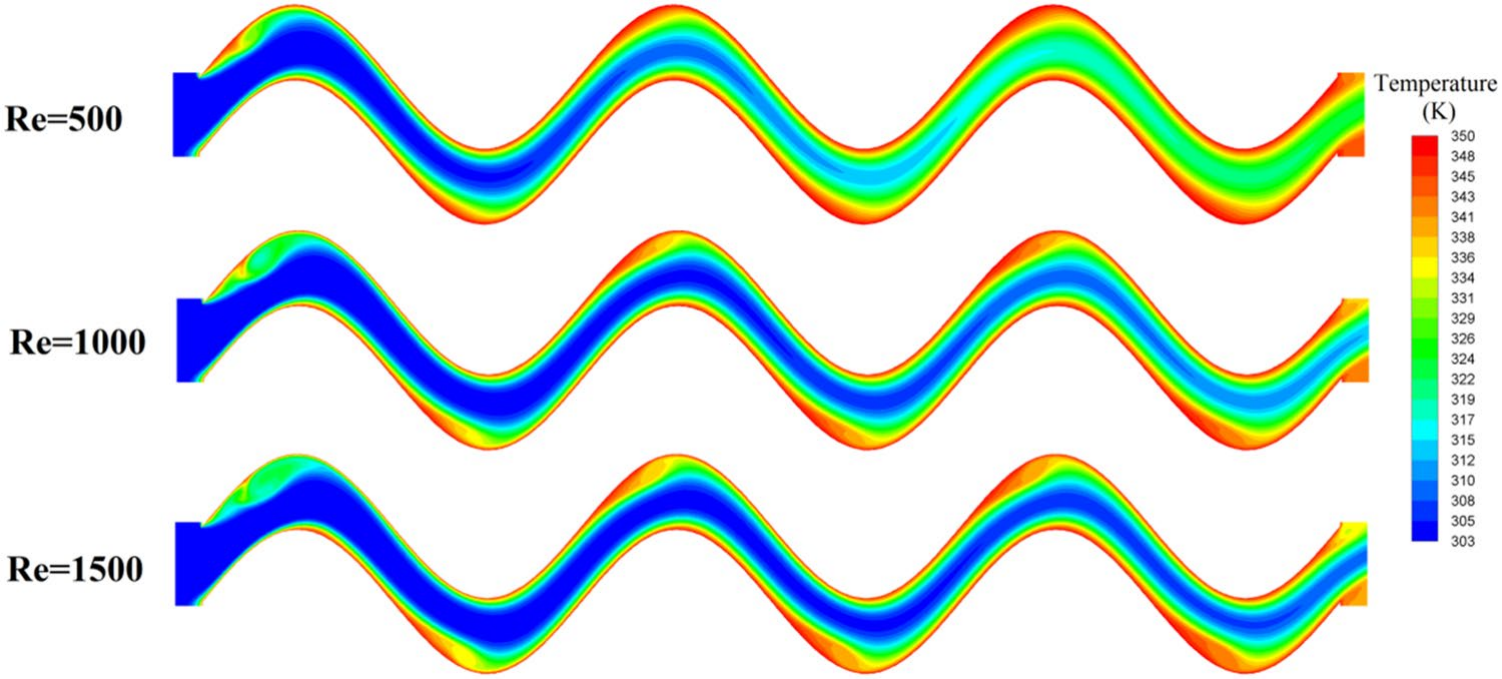
Temperature distribution of No.19 fin.

**Figure 16:**
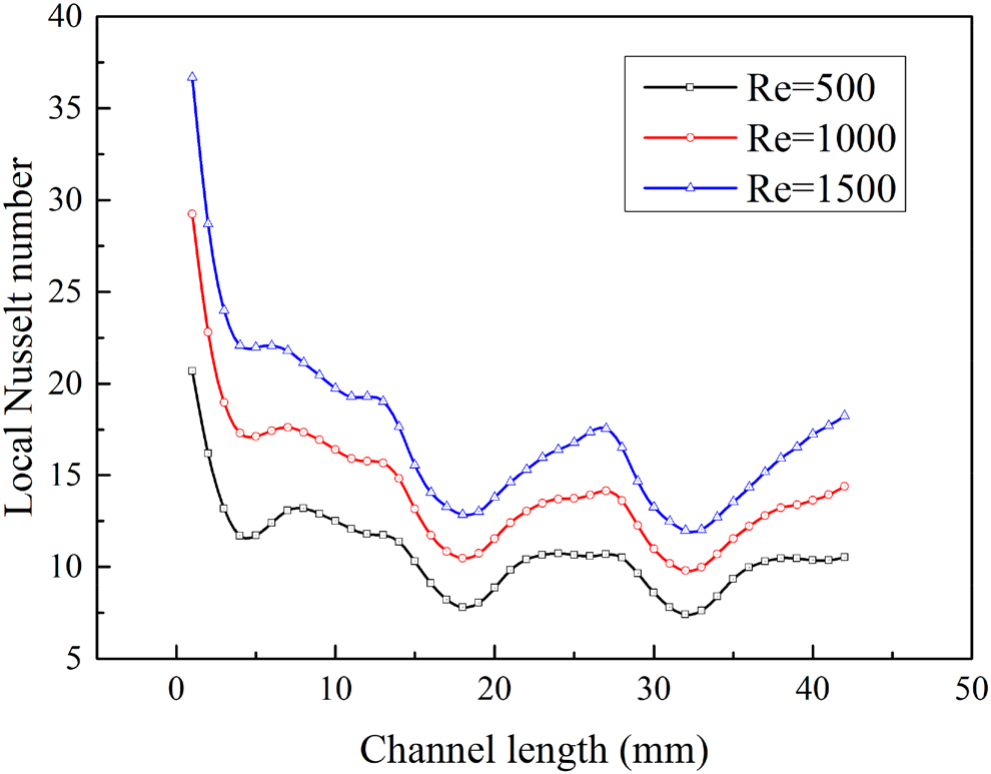
Local Nusselt number of No.19 fin.

The temperature field of No.19 Fin is shown in Fig. 15, and the cross-sectional position is *z* = 2.8mm. The air flow creates vortices at the peaks and troughs of the sinusoidal corrugated fins, while forming high temperature zones. The area of the high temperature region increases with the increase of Re, and the reflux in this area promotes convective heat transfer. The variation of the local Nusselt number Nu_x_ with flow direction is shown in Fig. 16. Nu_x_ increases with the increase of Re, and the variation range of Nu_x_ is 7.8 ~ 20.7 when Re = 500, and 11.9 ~ 36.7 when Re = 1500. Nu_x_ gradually decreases along the flow direction, because as the flow process progresses, the temperature difference between the air and the fins gradually decreases, and the convective heat transfer intensity gradually decreases. Nu_x_ in the first wavelength (x = 1 ~ 14mm) is higher, and Nu_x_ in the last two wavelengths is lower and similar in distribution.

## Conclusions

In this paper, a three-dimensional simulation model of SWFWF was established. The feasibility of the simulation model and calculation method was verified. Then, the heat transfer and pressure drop characteristic of SWFWF is studied by numerical method. 17 sets of fins of different sizes were designed, and the thermal–hydraulic characteristics of air-side flow were simulated, and empirical correlations were obtained on the basis of 140 data points. The conclusions are as follows:

1. When Re of air is constant, the j factor and the f factor show the same variation trend with the changes of *f*_p_/*f*_h_, *f*_h_/*W* and *2A*/*f*_p_, increase with the increase of parameters. While the parameters are constant, both the j factor and the f factor decrease as Re of air increases.
2. The dimensionless parameters have different effects on JF factors. When the values of *f*_p_/*f*_h_, *f*_h_/*W*, and *α* are 0.5, 0.4, and 70°, respectively, the *JF* factor has the maximum value of 1.18, 1.13, and 1.13 respectively. The optimized SWFWF simulation model is established, and the average *JF* factor is 1.307, which is about 10.9% higher than that of Fin 05 (JF = 1.18).
3. FSA shows a decreasing trend with the increase of *f*_p_/*f*_h_, *f*_h_/*W* and *2A*/*f*_p_. Among the calculated 17 sets of SWFWF, the minimum value of average FSA is 82.7°, when the value of *f*_*p*_*/f*_*h*_ is 0.5.
4. The empirical correlations of SWFWF are proposed. The mean deviation of the correlation of j and f factor are 2.5% and 7.2% respectively, and the average deviations are 0.85% and 4.9%, respectively. The correlations can be used for the design and optimization of SWFWF.

## Data Availability

The datasets used and/or analysed during the current study available from the corresponding author on reasonable request.

## List of symbols

*2A*: Fin amplitude (mm)
*A*_c_: Cross sectional area of flow channel (mm^2^)
*ΔC*_i_: The cell volume of the control volume (m^2^)
*c*_p,air_: Constant-pressure specific heat of air (J kg^−^^1^ K^−^^1^)
*d*_h_: Hydraulic diameter (mm)
*f*: Fanning friction factor
*f*_h_: Fin height (mm)
*f*_p_: Fin pitch (mm)
*f*_t_: Fin thickness (mm)
*h*_air_: Convective heat transfer coefficient of air (W m^−^^2^ k^−^^1^)
*j*: Colburn factor
*L*: Length of the fin (mm)
*L*_c_: Flow wetting perimeter (mm)
Nu: Nusselt number
Pr: Prandtl number
Re: Reynolds number
*u*: Velocity of air (m s^−^^1^)
*W*: Fin wavelength (mm)

## Greek symbols

*α*: Fin inclined angle (°)
Δ*P*: Pressure difference through fin core (Pa)
*θ*: The field synergy angle (°)
*θ*_i_: The field synergy angle of a single node (°)
*θ*_m_: The mean field synergy angle of the computational domain (°)
*λ*_air_: Heat transfer coefficient (W m^−^^1^ k^−^^1^)
*ν*: Kinematic viscosity of air (m^2^ s^−^^1^)
*ρ*_air_: Density of air (kg m^−^^3^)
*ρ*_m_: Means density (kg m^−^^3^)
